# Caudal Homeobox Protein Cdx-2 Cooperates with Wnt Pathway to Regulate Claudin-1 Expression in Colon Cancer Cells

**DOI:** 10.1371/journal.pone.0037174

**Published:** 2012-06-15

**Authors:** Ajaz A. Bhat, Ashok Sharma, Jillian Pope, Moorthy Krishnan, Mary K. Washington, Amar B. Singh, Punita Dhawan

**Affiliations:** 1 Department of Surgery, Vanderbilt University School of Medicine, Nashville, Tennessee, United States of America; 2 Department of Cancer Biology, Vanderbilt University School of Medicine, Nashville, Tennessee, United States of America; 3 Department of Pathology, Vanderbilt University School of Medicine, Nashville, Tennessee, United States of America; 4 Department of Medicine, Vanderbilt University School of Medicine, Nashville, Tennessee, United States of America; Aix-Marseille University, France

## Abstract

Dysregulation of tight junctions (TJs) is often associated with human diseases including carcinogenesis and recent studies support role of TJ integral proteins in the regulation of Epithelial-to-Mesenchymal Transition (EMT). In this regard, expression of claudin-1, a key constituent of TJs, is highly increased in colon cancer and is causally associated with the tumor growth and progression. However, mechanism/s underlying regulation of claudin-1 expression in intestinal epithelial cells remains poorly understood. In our studies, we have identified putative binding sites for intestinal transcription factors Cdx1, -2 and GATA4 in the 5′-flanking region of the claudin-1 gene. Our further studies using full length and/or deletion mutant constructs in two different human colon cancer cell lines, SW480 and HCT116, showed key role of Cdx1, Cdx2 and GATA4 in the regulation of claudin-1 mRNA expression. However, overexpression of Cdx2 had the most potent effect upon claudin-1 mRNA expression and promoter activity. Also, in colon cancer patient samples, we observed a significant and parallel correlation between claudin-1 and Cdx2 expressions. Chromatin immunoprecipitation (ChIP) assay confirmed the Cdx2 binding with claudin-1 promoter *in vivo*. Using Cdx2 deletion mutant constructs, we further mapped the Cdx2 C-terminus domain to be important in the regulation of claudin-1 promoter activity. Interestingly, co-expression of activated β-catenin further induced the Cdx2-dependent upregulation of claudin-1 promoter activity while expression of the dominant negative (dn)-TCF-4 abrogated this activation. Taken together, we conclude that homeodomain transcription factors Cdx1, Cdx2 and GATA4 regulate claudin-1 gene expression in human colon cancer cells. Moreover, a functional crosstalk between Wnt-signaling and transcriptional activation related to caudal-related homeobox (Cdx) proteins and GATA-proteins is demonstrated in the regulation of claudin-1 promoter-activation.

## Introduction

Tight junctions (TJs), the most apical cell-cell adhesion, help regulate the polarity and differentiated state of the epithelial cells. Disruption of cell-cell junctions along with concomitant changes in the expression of associated proteins helps induce invasion and metastatic progression in cancer. The claudin family of proteins is integral to the structure and function of tight junctions, and altered expression of claudin family members; although in a tissue specific manner, has been detected in multiple cancers. Several lines of evidence suggest that claudin-1 is a key constituent of TJs [1], [2], and is directly involved in the barrier and fence functions of TJs [3], [4]. Further, studies including ours have demonstrated that claudin-1 expression is dysregulated in a variety of cancers including colorectal cancer [3], [5]. We have further shown using a large cohort of colon cancer patients that claudin-1 mRNA expression is also highly increased in colon cancer [25]. It is further worthy here to note that in colon cancer, dysregulated claudin-1 expression associates with the tumor progression and metastasis [5]. Also, claudin-1 is a target of β-catenin/Tcf signaling, a key regulator of colonic homeostasis and neoplastic growth/progression [6]. However, details of the transcriptional regulation of colonic claudin-1 expression are not clearly understood.

**Figure 1:**
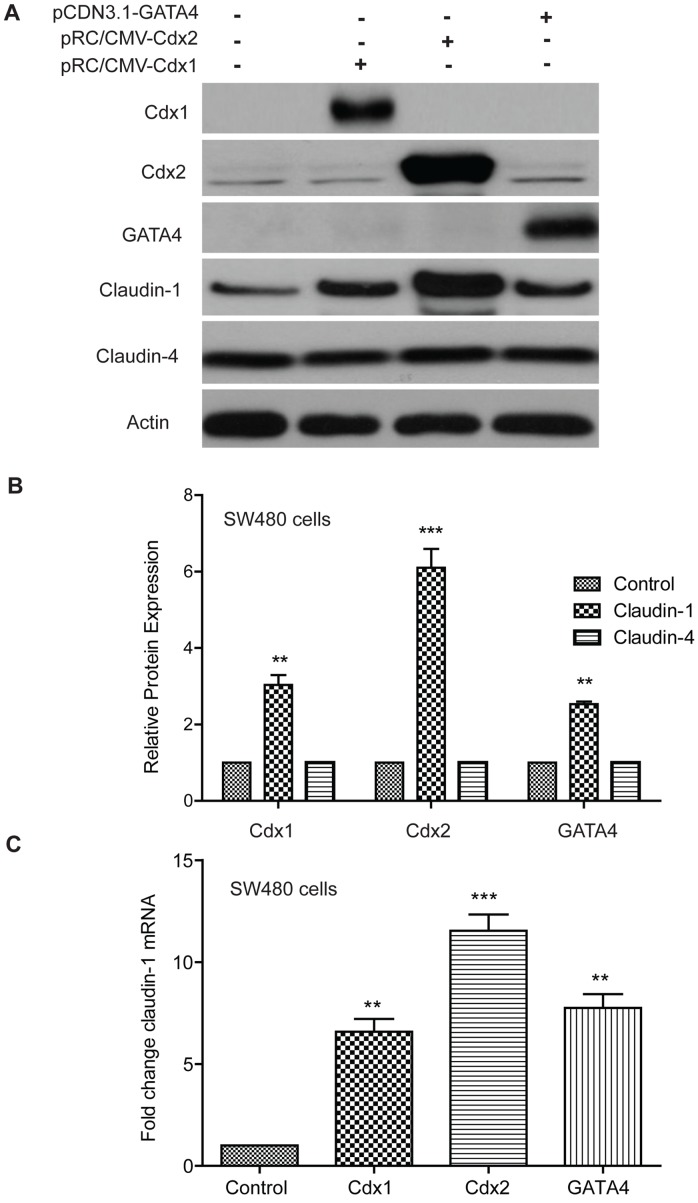
Effect of Cdx1, Cdx2 and GATA4 upon endogenous claudin-1 protein and mRNA expression in SW480 colon cancer cells. **A**. Western blot analysis of Cdx1, Cdx2, GATA4, claudin-1 and claudin-4 protein expression in SW480 cells. SW480 cells were transiently transfected with Cdx1, Cdx2 and/or GATA4 expression vectors, and 20 µg of whole cell extracts were analyzed. β-actin was used as loading control. **B**. Densitometry for claudin-1 and claudin-4 expression. **C**. Quantitative Real-Time PCR analysis. SW480 cells were co-transfected with Cdx1, Cdx2 and GATA4 expression vectors in the presence of the indicated reporter constructs, and quantitative RT-PCR was performed. Forty eight hours later, total RNA was isolated and subjected to real-time PCR using gene specific primers. Results were plotted as mean ± SD from three independent experiments and presented as fold change. *p<0.05, **p<0.01, ***p<0.001 when compared with control. Differences between two groups were analyzed by the two-tailed Student’s t-test and of more than two groups by one-way ANOVA with post-hoc Bonferroni Multiple Comparison test.

The homeodomain proteins Cdx1 and Cdx2 are the members of the caudal-related homeobox gene family [7]–[9]. Studies support the role of Cdx1 and Cdx2 in the regulation of early differentiation and maintenance of intestinal epithelial cells. Studies using epithelial cells of intestinal origin have demonstrated that modulation of the expression of Cdx1 and/or Cdx2 has significant functional effects on intestinal differentiation [10], [11], proliferation [10], [12], and intestine-specific gene transcription program [13]–[16]. It should be noted that Cdx1 and Cdx2 bind to several intestine-specific promoters to regulate intestine-specific gene transcription [13]–[15], [17], [18]. Also, GATA4, a zinc finger transcription factor, is involved in the regulation of proliferation and differentiation of intestinal epithelial cells and helps regulate embryonic development of the gastrointestinal tract [19]. Studies have further demonstrated that interactions of GATA4 with Cdx2 and HNF-1α help regulate expression of several cell-cell adhesion genes including E-cadherin and claudin-2 [19], [20].

In this report, we demonstrated that colonic claudin-1 gene expression is also a target of the transcriptional regulation by Cdx1, Cdx2 and GATA4 where Cdx2 appeared to have most potent effect as an inducer of colonic claudin-1 expression. We further provide data supporting parallel correlation between claudin-1 and Cdx2 expression in colon cancer patient samples. Most importantly, our data suggested that Cdx2 regulates claudin-1 expression in co-operation with Wnt-signaling, a known regulator of claudin-1 expression.

**Figure 2:**
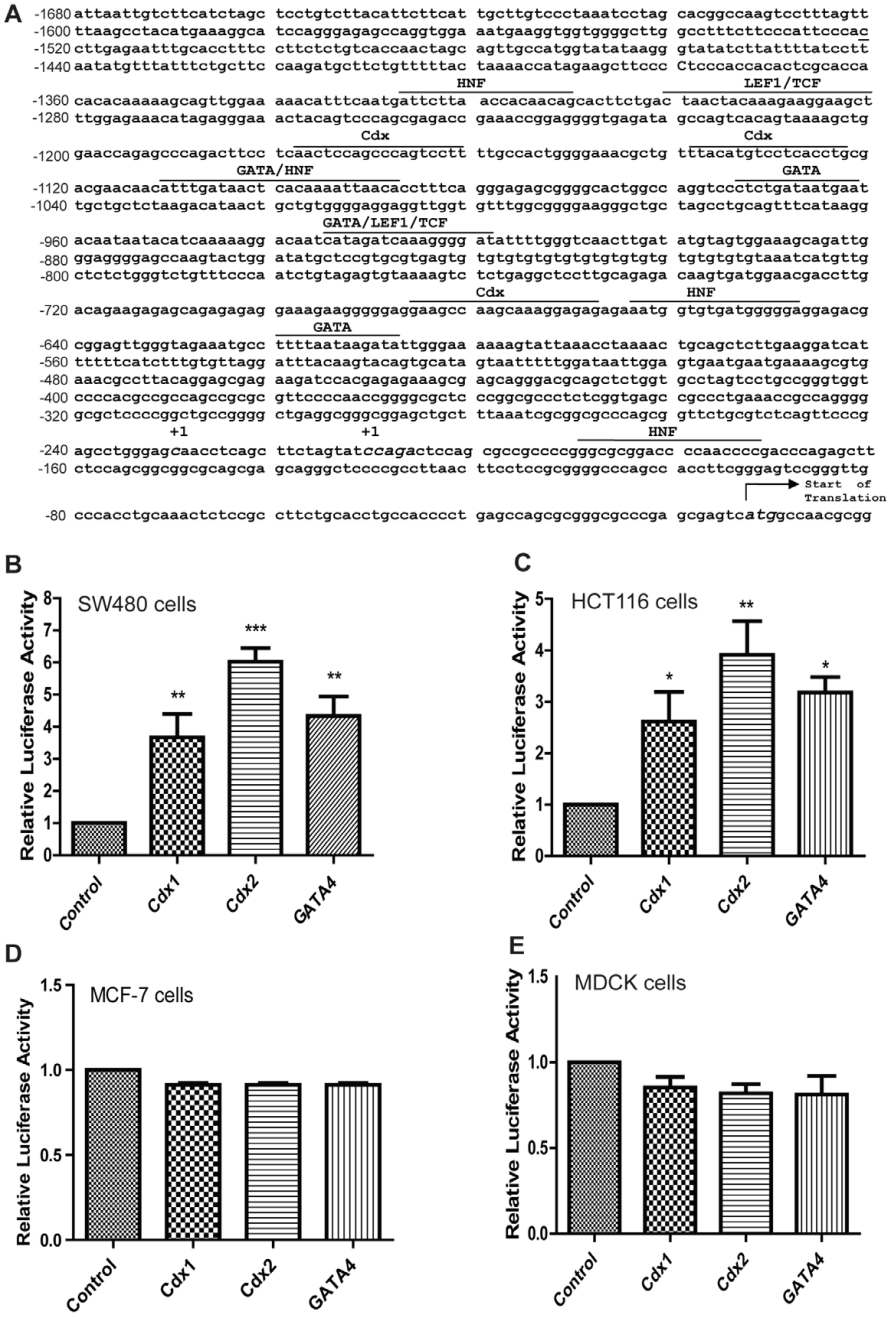
A. Sequence analysis of the 5′-flanking region of the human claudin-1 gene. The sequence of claudin-1 promoter and identification of the transcription-initiation site has previously been described [21]. Putative major consensus binding sites are underlined. **B.** Cdx1, Cdx2 and GATA4 regulation of claudin-1 luciferase reporter gene. SW480 (**B**), HCT116 (**C**), MCF-7 (**D**) and MDCK (**E**) cells were transiently transfected with a 1.2-kb claudin-1-luciferase reporter plasmid along with Cdx1, Cdx2 and/or GATA4 expression vectors. Empty pGL3-basic vector was used for control purposes. Nonspecific plasmid DNA was used to maintain equal amounts of DNA in all transfection samples. Results are expressed in fold-activation of relative luciferase activity after normalization with *Renilla* activity from 3 independent experiments, and the values are expressed as means ± SD. *p<0.05, **p<0.01, ***p<0.001 when compared with control. Differences between two groups were analyzed by the two-tailed Student’s t-test and of more than two groups by one-way ANOVA with post-hoc Bonferroni Multiple Comparison test.

## Results

### Forced Expression of Cdx1, Cdx2 or GATA4 Induces Claudin-1 Expression in Colon Cancer Cells

To examine whether Cdx1, Cdx2 and/or GATA4 help regulate colonic claudin-1 expression, we determined the effect of over-expression of above transcription factors upon endogenous claudin-1 expression. For this purpose, we used SW480 colon cancer cells, which express low levels of endogenous claudin-1 [5]. Mammalian expression plasmid constructs containing full-length Cdx1, Cdx2 or GATA4 were transiently transfected in SW480 cells. Empty expression plasmids served as control. Forty-eight hours post-transfection, samples were collected and were subjected to the preparation of lysate or total RNA extract. Immunoblotting using antigen-specific antibody was done to confirm overexpression and similar levels of overexpression of antigens under study (Figure 1A). As shown in Figure 1B, we observed robust upregulation of claudin-1 in cells overexpressing Cdx1 (3.8 fold±0.451), Cdx2 (6.5 fold±0.854) or GATA4 (3.3 fold±0.115) at protein level. Quantitative RT-PCR showed similar induction of claudin-1 mRNA expression upon overexpression of all the above transcription factors and once again this induction was maximum in cells over-expressing Cdx2 (Figure 1C). This effect of Cdx1, Cdx2 or GATA4 upon claudin-1 expression was specific because expression of claudin-4, yet another claudin family member, was not affected by the forced expression of any of the above transcription factors (Figure 1A & B).

**Figure 3:**
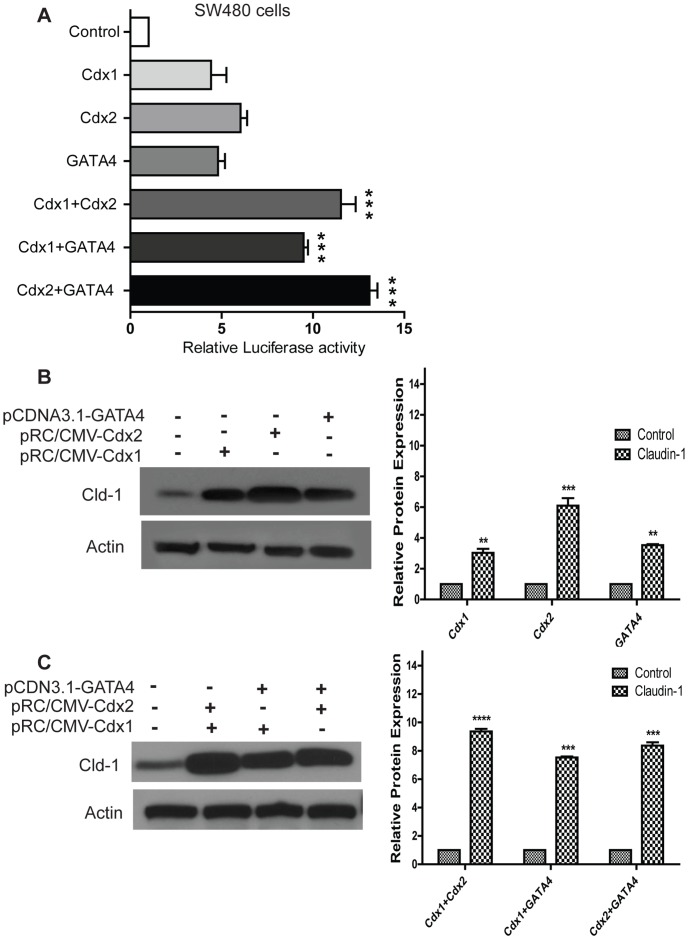
A. Synergistic effect of Cdx1, Cdx2 and GATA4 overexpression upon claudin-1 protein expression and gene transcription. SW480 cells were co-transfected with different combinations of Cdx1, Cdx2 and GATA4 expression vectors in the presence of indicated reporter constructs, and luciferase assays were performed. Forty eight hours later, cells were lysed and luciferase activity was measured. Results were plotted as mean ± SD from three independent experiments and normalized luciferase activity in each cell line was presented as relative luciferase activity. The mean value of cells transfected with pGL3-basic vector in the absence of expression vectors was set to 1. **B** and **C**. Western blot analysis of claudin-1 protein expression in SW480 cells. SW480 cells were transfected with different combinations of Cdx1, Cdx2 and GATA4 expression vectors and 20 µg of proteins were analyzed. **p<0.01, ***p<0.001, ****p<0.0001 when compared with control. Differences between two groups were analyzed by the two-tailed Student’s t-test and of more than two groups by one-way ANOVA with post-hoc Bonferroni Multiple Comparison test.

**Figure 4:**
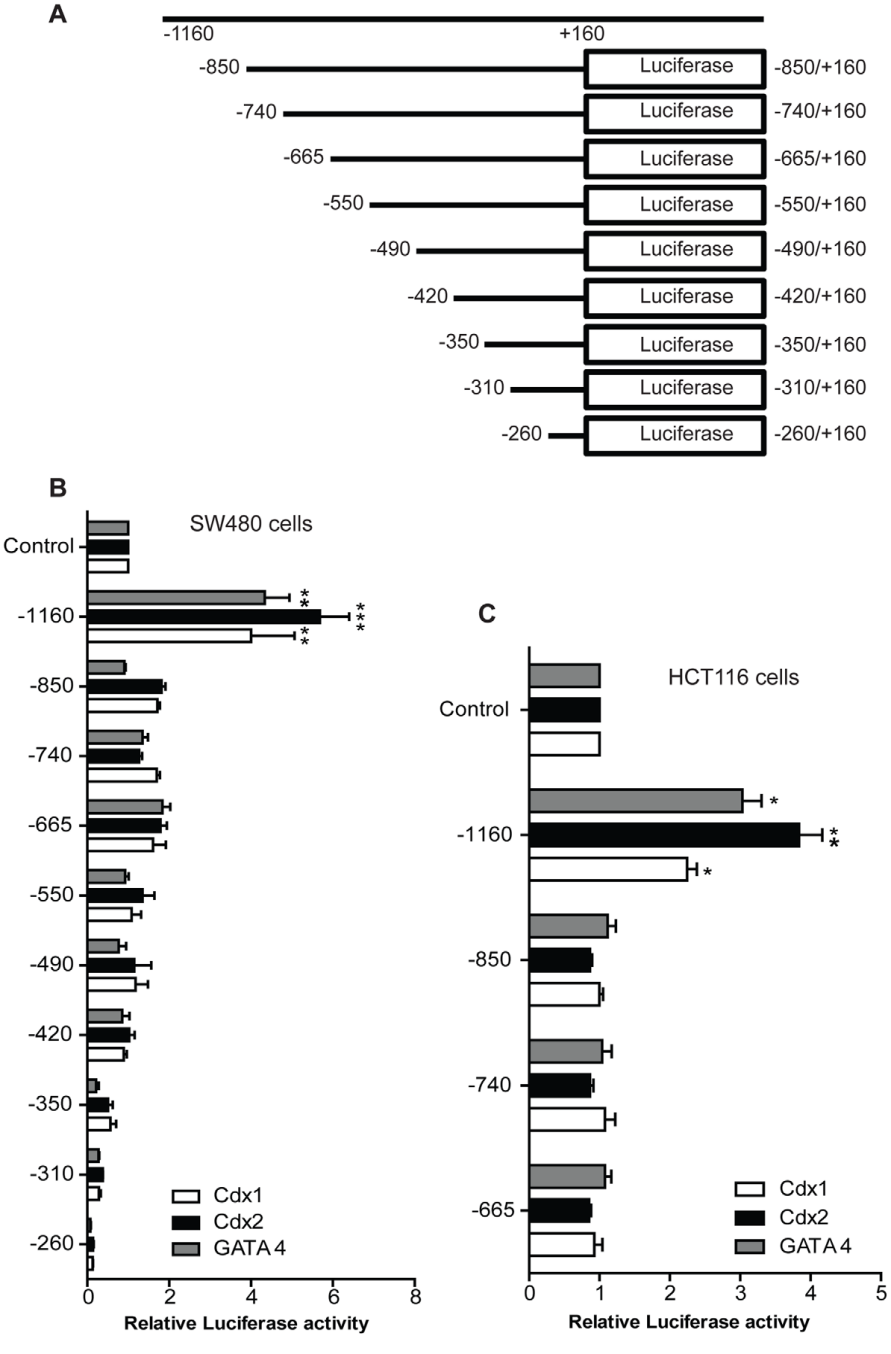
Analysis of human claudin-1 promoter deletion constructs. **A**. The reporter constructs containing sequentially deleted 5′- flanking fragments of the 1.2 kb claudin-1(–1160/−260) were prepared and transfected into SW480 (**B**) and HCT116 (**C**) cells as described under “Experimental Procedures”. The results are expressed as relative luciferase activity of three different experiments carried out in triplicate (mean ± S.D.). Differences between two groups were analyzed by the two-tailed Student’s t-test and of more than two groups by one-way ANOVA with post-hoc Bonferroni Multiple Comparison test.

### Cdx1, Cdx2 and GATA4 Regulate Claudin-1 Gene Transcription in Colon Cancer Cells

In above studies, forced expression of Cdx1, Cdx2 or GATA4 resulted in an increase in claudin-1 protein and mRNA expressions. Therefore, we further determined whether Cdx1, Cdx2 and/or GATA4 help regulate claudin-1 transcription. In this regard, we performed luciferase reporter assay using a human claudin-1 promoter (–1160/+160)–luciferase reporter construct described before [21] referred as pGL3/−1.2Cld-1 (Figure 2A) throughout the manuscript. The -1160 to +160 bp region of human claudin-1 promoter contains putative Cdx, HNF and GATA binding sites along with the putative binding sites for TCF/Lef-1 (Figure 2A). SW480 cells were transiently transfected with the pGL3/−1.2Cld-1 or pGL3-basic (vector alone) along with a reference reporter plasmid. The luciferase activity of pGL3/−1.2Cld-1 was normalized to that of pGL3-basic in addition to the reference vector. Ectopic expression of Cdx1 or GATA4 did not alter the expression level of Cdx2, nor did Cdx2 overexpression have a noticeable effect upon Cdx1 or GATA4 protein expressions (Figure 1A). Over-expression of either Cdx1 or GATA4 resulted in a ∼3-fold increase in the promoter activity of pGL3/−1.2Cld-1. However, similar over-expression of Cdx2 resulted in a 6-fold increase in the activity of the same construct (Figure 2B). We observed a similar trend of induction of pGL3/−1.2Cld-1-driven luciferase activity in HCT116 colon cancer, which was 2.5–3 fold upon overexpression of Cdx1 and GATA4 and ∼4-fold upon overexpression of Cdx2 (Figure 2C). We obtained similar outcome using a longer 3.3 Kb (-3140 to +160) claudin-1 promoter reporter assay, which was not significantly different compared to the 1.2 Kb claudin-1 promoter (Figure S1). Therefore, we continued the use of the 1.2 Kb claudin-1 promoter for further studies. Our further studies supported specificity of our above findings as over-expression of yet another transcription factor HNF-1α did not affect claudin-1 promoter activity in either SW480 cells (Figure S2). We further confirmed specificity of our findings to the colonic claudin-1 expression as similar transient overexpression of Cdx1, Cdx2 or GATA4 in human breast cancer cells (MCF-7) or renal epithelial cells (MDCK II cells) did not increase claudin-1 promoter activity (Figure 2D & E).

**Figure 5:**
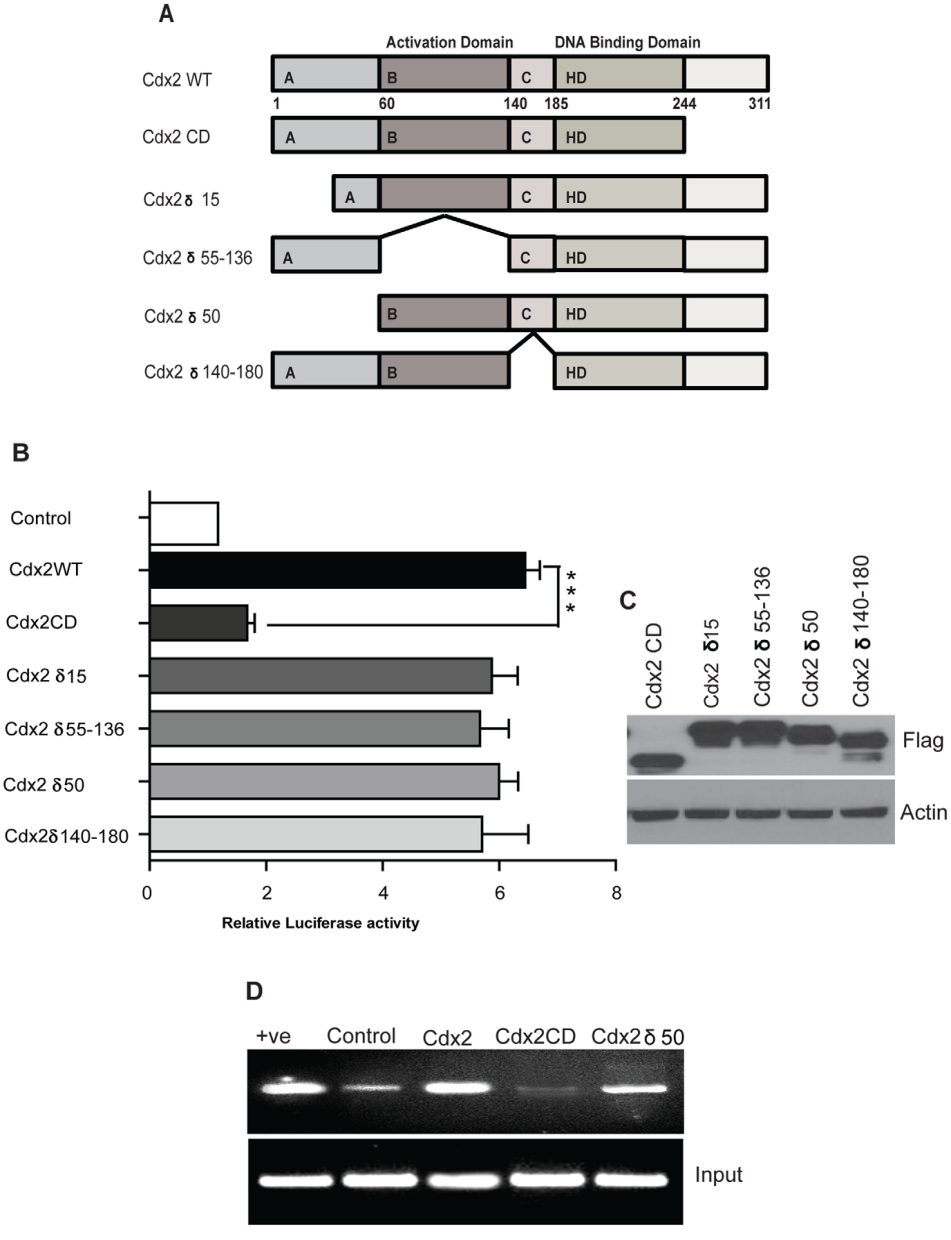
A. Cdx2 deletion mutants. HD: homeodomain; A, B, C: conserved domains. **B**. Luciferase activity after co-transfection with Cdx2 wild-type and truncation mutants. Deletion of the Cdx2 C-terminus (Cdx2CD) significantly (***p<0.001) reduced claudin-1 reporter activity. **C**. Western blot analysis using anti-Flag antibody to check equivalent expression of Cdx2 truncation mutants in SW480 cells transiently transfected with Cdx2 truncation mutant expression vectors. β-actin was used as loading control. **D**. Cdx2 binding to the 5′-flanking region of claudin-1 gene promoter shown by ChIP assay. Formaldehyde cross-linked chromatin prepared from 5×10^7^ SW480 cells was incubated with antibodies to Cdx2 or with IgG (negative control). Cross-linking of the immunoprecipitates was reversed and the DNA purified and analyzed using primers specific for either the claudin-1 promoter region or for glyceraldehyde-3-phosphate dehydrogenase. Results show that the induction of claudin-1 promoter activity by Cdx2 requires Cdx2-C-terminus domain.

Cdx1, Cdx2 and GATA4 transcription factors work synergistically to regulate expression of different genes [19]. Therefore, we determined whether a similar synergism existed amongst Cdx1, Cdx2 and GATA4 in the regulation of claudin-1 gene transcription. To test, we co-transfected Cdx1 with Cdx2, Cdx1 with GATA4 and Cdx2 with GATA4 in SW480 cells. Indeed, co-expression of Cdx1 with Cdx2, Cdx1 with GATA4 or Cdx2 with GATA4, all resulted in significant increase (9–13 fold) in claudin-1 promoter activity (Figure 3A). This synergism in the regulation of claudin-1 expression was further confirmed at the protein levels where claudin-1 expression was induced (8–10 fold) as compared to (3–6 fold) with Cdx1, Cdx2 or GATA4 alone (Figure 3B & C).

**Figure 6:**
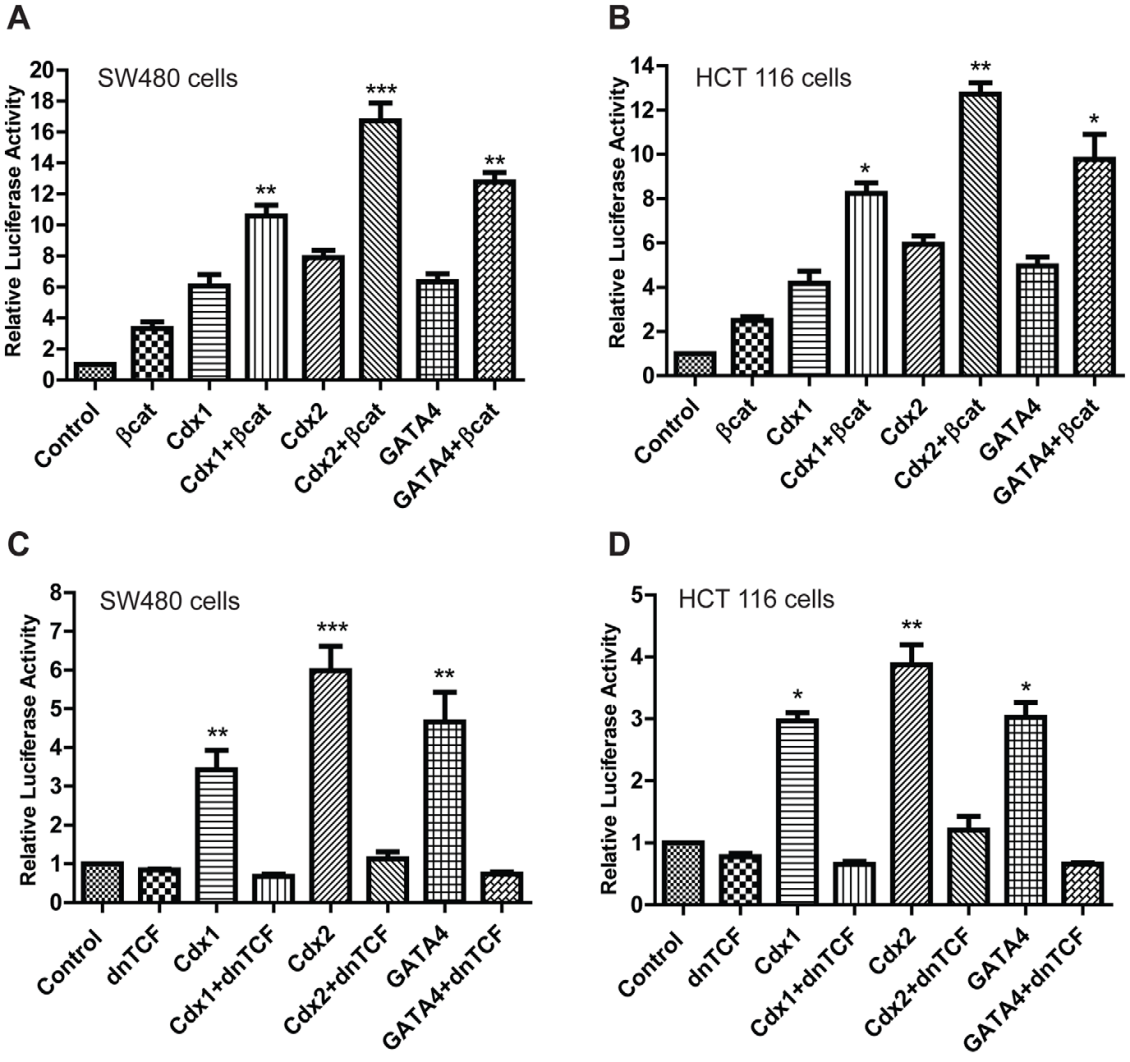
Wnt-signaling helps mediate Cdx, GATA-dependent claudin-1 gene expression. Claudin-1 promoter activity in SW480 (**A**) and HCT 116 cells (**B**). Cells were transiently co-transfected with expression vector constructs for activated β-catenin (S33Y) along with Cdx1, Cdx2 or GATA4 expression vectors. Claudin-1 promoter activity was abrogated upon co-transfection of dnTcf-4 with Cdx1, Cdx2 or GATA4 expression vectors SW480 (**C**) and HCT116 (**D**) cells.

### Claudin-1 Promoter Contains Binding Sites for Cdx and GATA Transcription Factors

Based upon our above findings, we further examined putative binding sites for Cdx1, Cdx2 and GATA4 in claudin-1 promoter. We used computational analysis using MatInspector software (Oxford Molecular Group) to identify the putative binding sites for consensus Cdx- and GATA-binding elements (Figure 2A). To confirm the functional efficacy of these putative binding sites and also to identify the regions involved in the regulation of claudin-1 gene transcription, we generated claudin-1 promoter deletion constructs starting from the 5′-flanking region as described previously [21]. Figure 4A depicts the full length promoter construct (-1160/+160) and nine deletion constructs (−850/+160, −740/+160, −665/+160, −550/+160, −490/+160, −420/+160, −350/+160 and −260/+160). The reporter constructs generated above were then co-transfected with Cdx1, Cdx2 or GATA4 expression constructs into SW480 and HCT116 cells. The full-length promoter construct contained the consensus binding sites for Cdx and GATA4, along with the binding site for Tcf/Lef-1, and induced claudin-1 promoter activity 5–6 fold in SW480 cells (Figure 4B). However, this induction was lost upon deletion of the proximal 310 bp (−1160 to −850), which suggested that this 310 bp region has sites important for claudin-1 promoter induction by transcription factors under investigation. Similar findings from HCT116 cells supported this notion (Figure 4C). Both cell lines exhibited similar patterns of promoter activity although signals in general were stronger in SW480 cells. This 310 bp region contained a Cdx, two GATA consensus binding sites and a Tcf/Lef-1 binding site suggesting potential importance of these sites in the regulation of claudin-1 gene expression.

**Figure 7:**
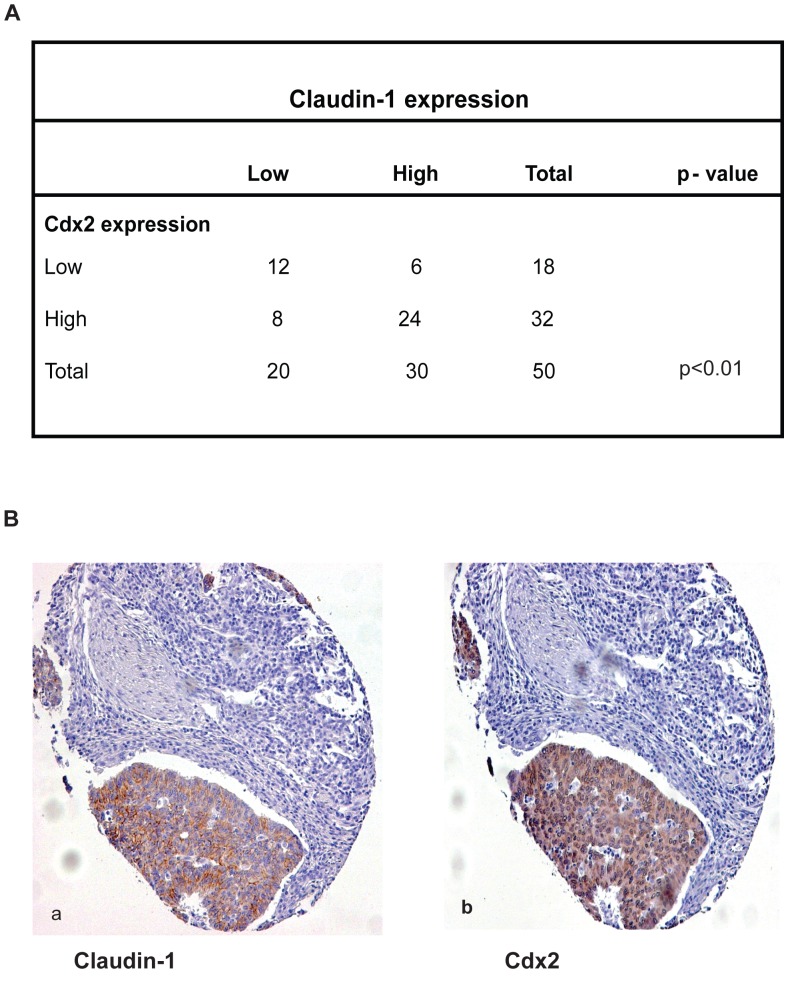
Correlation between claudin-1 and Cdx2 expression in colon cancer samples. **A**. Correlation between claudin-1 and Cdx2 expressions in colon cancer. Data from custom colorectal cancer tissue arrays (tissue microarrays developed at Vanderbilt) that contain surgical specimens from 50 primary and paired metastatic fresh-frozen human colorectal cancer samples are shown. A significant direct correlation between claudin-1 and Cdx2 expression was observed (**P<0.01) using Yates’s chi square test. **B**. representative staining for claudin-1 (**a**) and Cdx2 (**b**) in colon cancer patient samples. Sections were counterstained with hematoxylin to determine the structural details.

### The C-terminal Domain is Required for Cdx2 Mediated Activation of Claudin-1 Transcriptional Activity

Above studies suggested a role of Cdx1, Cdx2 and GATA4 in the regulation of claudin-1 promoter. However, Cdx2 appeared to have the most potent effect upon claudin-1 promoter activity. Therefore, in further studies, we determined the Cdx2 domain that is required for the transcriptional activation of claudin-1 promoter. In this regard, we tested the effect of Cdx2 deletion mutant constructs for their ability to induce claudin-1 promoter activity (Figure 5A) [22]. Immunoblot analysis using anti-flag antibody confirmed similar expression levels of Cdx2 deletion mutant constructs (Figure 5C). Results showed that deletion of the canonical transcription activation domain (Cdx2 δ55–136), the N-terminal domains (Cdx2 δ15 or Cdx2 δ50) or the region between amino acids 140 and 180 (Cdx2 δ140–180) had no significant effect on claudin-1 promoter activity. In contrast, deletion of the Cdx2 C-terminus 244–311 (Cdx2CD) reduced claudin-1 promoter activity by 5-fold compared to wild type Cdx2 (Figure 5B). Taken together, these studies suggest that Cdx2 C-terminus domain is required for the Cdx2-dependent induction of claudin-1 promoter activation.

To further determine if Cdx2 binds to the claudin-1 promoter *in vivo*, we performed ChIP analysis and compared SW480 cells overexpressing Cdx2 with SW480 cells transfected with the empty expression plasmid vector. The Figure 5D shows the resulting DNA pulled down by anti-Cdx2 antibody containing the sequence flanking the Cdx binding site. This suggested that Cdx2 binds to the Cdx-sequence elements within the 310 bp of claudin-1 promoter and this binding increases upon overexpression of Cdx2 in SW480 cells, which, in turn, increases claudin-1 promoter activity. To further determine if C-terminal is important for Cdx2 binding to claudin-1 promoter, we over-expressed Cdx2CD (C-terminal deletion mutant) or an N- terminal deletion mutant that does not affect the claudin-1 transcriptional activity (Cdx2 δ50) in SW480 cells and performed ChIP analysis. The ChIP analysis demonstrated that the increased binding due to full-length Cdx2 overexpression was abrogated upon overexpression of Cdx2CD but not the N-terminal deletion (Cdx2 δ50) mutant (Figure 5D). These findings suggested that Cdx2 C-terminal domain is important for the binding of Cdx2 to claudin-1 promoter and Cdx2-dependent induction of claudin-1 promoter activity.

### A Functional Crosstalk of Wnt Signaling and Cdx-related Transcriptional Activation Regulates Claudin-1 Promoter Activity

Claudin-1 is a known target of Tcf/Lef-1 signaling [6] and Tcf/Lef-1 elements are present within the −1160 to −850 region of claudin-1 promoter. To test the effect of β-catenin/Tcf/Lef-1, claudin-1 promoter was subjected to co-transfection in presence or absence of activated β-catenin (S33Y) along with Cdx1, 2 or GATA4. Transient transfections were done in SW480 and HCT116 cells and luciferase activity of pGL3/−1.2Cld-1 was normalized to pGL3-Basic in addition to the reference vector. Overexpression of activated β-catenin (S33Y) with Cdx1, Cdx2 or GATA4 resulted in atleast two fold further induction of claudin-1 promoter-mediated gene expression compared to Cdx1, Cdx2 or GATA4 alone (Figure 6A &B). Conversely, when we over-expressed Cdx1, Cdx2 or GATA4 with dnTcf-4, the induction of claudin-1 promoter was abrogated suggesting potential cross talk between these factors (Figure 6C &D). Taken together, our data suggested that modulation of β-catenin/Tcf/Lef activity plays central role in the Cdx, GATA4 mediated regulation of claudin-1 expression.

### Claudin-1 and Cdx2 Expressions are Correlated in Human Colorectal Carcinoma Samples

Cdx2 protein expression is associated with advanced stages of colon tumorigenesis [23]. Claudin-1 expression positively correlates with tumor progression and metastasis in colon cancer [5]. Therefore, we investigated a possible correlation between Cdx2 and claudin-1 expressions in colon cancer. We examined the potential correlation between claudin-1 and Cdx2 expressions in a large population of human tissue samples. Immunohistochemical analysis for claudin-1 and Cdx2 in custom colorectal cancer tissue arrays (tissue microarrays developed at Vanderbilt University by Dr. M.K. Washington; which contain surgical specimens from 50 primary and paired metastatic fresh frozen human colorectal cancer sample) was performed. Results showed predominant nuclear immunostaining for Cdx2 while claudin-1 staining was predominantly cytosolic though some punctate membrane staining was also observed (Figure 7B). Importantly, further analysis demonstrated a significant correlation (p<0.01) between claudin-1 and Cdx2 expressions in 36∶50 (72%) colon cancer samples (Fig. 7A). Taken together, these data suggest a direct correlation between the expressions of Cdx2 and claudin-1 in colorectal carcinomas.

## Discussion

Dysregulation of colonocyte differentiation lies at the core of colon carcinogenesis. Claudin-1 expression is highly increased in colon cancer and is causally associated with the tumor growth and progression [5]. Of interest, in addition to the dysregulated protein expression and localization, claudin-1 mRNA expression also increases in colon cancer [25]. It is also worthy to note that genetic manipulation of claudin-1 expression in colon cancer cells resulted in sharp and inverse changes in their differentiation status including E-cadherin expression [5], [24]. Also, induction of epithelial differentiation in colon cancer cells using Histone deacetylase (HDAC) inhibitors associated with a specific decrease in claudin-1 expression among claudin family members [25]. Taken together, claudin-1 expression appears to affect colon carcinogenesis through the modulation of colonic epithelial cell differentiation. Therefore, we examined the role of transcription factors important in the regulation of intestinal epithelial differentiation as well as colon carcinogenesis in the regulation of claudin-1 mRNA transcription. The key outcome of our studies using two different and widely used colon cancer cell lines were (i) Cdx1, Cdx2 and GATA4 help regulate claudin-1 gene transcription and this regulation was specific to the cells of colonic origin, (ii) Cdx2 is the most potent inducer of claudin-1 promoter activity amongst Cdx1, Cdx2 and GATA4, and physically binds to the Cdx-consensus binding site/s in claudin-1 promoter, as determined by the ChIP analysis, (iii) Cdx1, Cdx2 and GATA4 regulate colonic claudin-1 expression through potential synergism among each-other and through the potential cross-talk with the Wnt-signaling pathway. Taken together, in this report we provide the first insights into the complex transcriptional activation events, which regulate colonic claudin-1 expression.

The caudal homeobox protein Cdx1 is expressed mainly in the crypt compartment, whereas the Cdx2 protein is detected in both the crypt and the villus compartments [26]. Importantly, outcome from multiple studies suggest that the role of Cdx1 and Cdx2 in the regulation of intestinal homeostasis is often complementary and may depend upon the maturation of the intestinal epithelial cells [27]. These findings are complemented with the demonstration of a rather discrete role of Cdx1 in the regulation of intestinal epithelial cell proliferation, while Cdx2 play important role in the regulation of the differentiation status of the intestinal epithelial cells [28]. GATA4 also belongs to a family of transcription factors that play important role in the regulation of embryogenesis and possibly early maturation of intestinal epithelial cells [29]. GATA4 along with GATA5/6 has been associated with cell survival, cell proliferation, and neoplastic transformation of various cell types [8]–[15]. Notably, among gastrointestinal cancers, GATA4 is amplified in ∼10% of esophageal adenocarcinomas and Barrett metaplasia [30]. Role of GATA4 in the regulation of colon carcinogenesis is not well studied though limited studies suggested that it may function as a tumor suppressor in the colon cancer [31]. However, one need to be cautioned in the specific functional formulation of such transcription factors as their function/s are often context dependent and interaction with other transcriptional regulators are also important.

Of interest, role of Cdx2 transcription factor in the regulation of colon carcinogenesis remain controversial and studies suggest its role as a positive or negative ‘tumor modulator’. In this regard, mice that are heterozygous-null for Cdx2 gene develop colonic hamartomas after the remaining Cdx2 allele is lost [32], [33]. These heterozygous-null Cdx2 mice are also sensitive to the azoxymethane (AOM)-induced colonic adenocarcinoma [34]. Also, the compound heterozygotes for Cdx2 and Adenomatous Polyposis Coli (Apc) genes develop more adenomatous polyps in the colon compared to their heterozygous *Apc* littermates [35]. Together, above findings would suggest function of Cdx2 as a colon tumor suppressor. However, immunohistochemical analysis has detected strong Cdx2 expression in ∼90% of the human colon cancer samples [38], [39]. Further, Cdx2-overexpression in colon cancer cells induces anchorage-independent growth and cell survival [36]. Of interest, Cdx2 promotes anchorage-independent growth through the transcriptional repression of IGFBP-3 [37]. Thus, a tumor-promoting role of Cdx2 in colon cancer can be envisaged. Taken together, it can be postulated that the role of Cdx2 in the regulation of colon cancer may depend upon the differentiation status of the colon cancer cells and interaction with other transcription factors.

Findings from multiple studies have unveiled existence of a complex interaction among Cdx1, Cdx2, GATA4 and HNF-1α in the induction of intestinal gene expression including claudin family member, claudin-2 [40], [41]. It is important to note that similar to claudin-1, claudin-2 expression is also upregulated in colon carcinogenesis [42]. Cdx2 has been demonstrated to play role in the regulation of other cell-cell adhesion proteins including LI-cadherin, claudin-3 and claudin-4 [20]. In our current study, we have identified consensus Cdx-binding site as well as GATA-binding site in the human claudin-1 promoter. The fact that forced expression of Cdx1, Cdx2 or GATA4 induced not only claudin-1 protein and mRNA expression but also induced luciferase-reporter activity dependent upon the full-length human claudin-1 promoter (−1160 to +160) supports a causal role of above transcription factors in the regulation of claudin-1 expression. Notably, deletion of the claudin-1 promoter sequence from −1160 to −840 bp (310 bp deletion) of the 5′-flanking region containing the Cdx- and GATA4 binding sites abrogated the increase in the luciferase activity observed using the full-length promoter construct. Further, our finding that the Cdx1, Cdx2 or GATA4-dependent induction of claudin-1 promoter activity can be further enhanced when above transcription factors were co-expressed supports a complex inter-dependence among these transcription factors in the regulation of claudin-1 expression. The fact that similar forced expression of HNF-1α did not affect claudin-1 expression renders specificity to our findings (Figure S2). Further, our finding that similar overexpression of Cdx1, Cdx2 or GATA4 in the renal epithelial and breast cancer cells had no apparent effect upon claudin-1 expression suggest that this is not a universal mechanism of claudin-1 expression and is rather colon specific. It is possible that these factors may require other co-factors to be active, which are present only in the colon cancer cells.

Our data further suggested that among the transcription factors under investigation, Cdx2 is the most potent inducer of claudin-1 expression. Indeed, our studies using ChIP analysis demonstrated a direct association of the Cdx2 with claudin-1 promoter and thus suggested that claudin-1 is a direct target for Cdx2-dependent transcriptional regulation. It is further noteworthy that observed binding of Cdx2 with claudin-1 promoter was not dependent upon Cdx2-transcription activation domain as deletion of the Cdx2 N-terminus (Cdx2ND) had no effect upon the Cdx2 binding with claudin-1 promoter. Interestingly, our studies demonstrated that the carboxyl-terminus of Cdx2 is required for the association of Cdx2 with claudin-1 promoter as ChIP analysis using a Cdx2 C-terminal mutant (Cdx2CD) completely abrogated the increase in the binding of Cdx2 to claudin-1 promoter that was induced upon expression of full-length Cdx2. This finding is interesting as previous studies have suggested that DNA binding domain of Cdx2 spans from 180–244 amino acids, compared to its C-terminal (244–311 amino acids) which appeared critical for the binding of Cdx2 to claudin-1 promoter as well as induction of claudin-1 promoter activity in our current study. Taken together, we speculate that the C-terminus of Cdx2 protein may directly/indirectly help modulate Cdx2 binding to the DNA. Our data regarding the importance of C-terminus in Cdx2-dependent transcriptional regulation is well supported by a previous study where deletion of the Cdx2 C-terminus (Cdx2CD) also reduced sucrase isomaltase reporter activity by ∼50% in colon cancer cells [22]. However, further studies will be needed to clearly understand the details of the mechanism underlying Cdx2 binding with claudin-1 promoter and are currently underway in our laboratory.

We further documented a novel finding that the Cdx1, Cdx2 and/or GATA4-dependent increase in claudin-1 promoter activity was completely abolished when Wnt-signaling was inhibited using co-transfection of the dn-Tcf construct. Conversely, effects of Cdx1, Cdx2 or GATA4 expression upon claudin-1 promoter was further induced upon simultaneous activation of Wnt-signaling, using expression of the activated β-catenin (S-33Y β-catenin) mutant construct. Overall, these findings suggested a cross-talk between β-catenin/Tcf/Lef-1 and Cdx1, Cdx2 and/or GATA4 in the regulation of claudin-1 mRNA transcription. Notably, Wnt-signaling is known to regulate the expression as well as biological actions of the homeodomain transcription factors [43]–[45]. A recent study has also demonstrated that Cdx2 binds many of the same *cis*-regulatory regions as Tcf-4 in the intestinal epithelial cells, and the Tcf-4 and Cdx2 co-occupancy predicts intestinal gene expression better than occupancy by either of the factor alone [46]. Claudin-1 itself is a known target of Tcf/Lef-dependent transcription regulation [6]. Taken together, we postulate that co-occupancy by a Wnt-effector protein and a tissue-restricted homeobox transcription factor may represent the means for a tissue-specific response in the intestinal context, including increased colonic claudin-1 expression. Further, a functional crosstalk between tight junction and adherens junction constituents has been demonstrated [47], [48]. Thus, it can be postulated that the β-catenin-Lef/Tcf-dependent regulation of claudin-1 gene expression is part of the signaling mechanism/s functionally relevant to the oncogenic transformation. Such a notion gains support by published findings from ours and other laboratories that increased claudin-1 expression in human colon cancer cells bears causal correlation with the APC mutation [5], [6]. Taken together, the co-existence of the binding sites for Lef/Tcf and Cdx-proteins in claudin-1 promoter suggests an interacting network of direct and indirect modulation of claudin gene expression by homeodomain transcription factors along with Wnt-signaling pathway.

In summary, our experimental outcome support a model in which claudin-1 expression is governed by a complex interaction/inter-dependence among Cdx1, Cdx2 and GATA4 and a functional cross-talk with the Wnt/Tcf/Lef-1 activity in an organ specific manner. Our data supporting a significant and parallel correlation between claudin-1 and Cdx2 expression in colon cancer patient samples highlights the importance of this regulatory relationship in the regulation of colonic homeostasis. Further studies are needed to understand how above described regulatory mechanism functions in the normal colonic epithelial cells vs. cancer cells. Irrespective, findings from our current studies demonstrates a complex regulation of claudin-1 expression, which is critically relevant to the barrier function of normal epithelial cells and oncogenic potential of the colon cancer cells. We believe that our studies may shed new light in a potential causal association of claudin-1 in the regulation of colonic epithelial differentiation and its deregulation during the neoplastic growth and oncogenic transformation.

## Materials and Methods

### Plasmids and Reagents

The antibodies against claudin-1 and -4 were from Invitrogen (San Francisco, CA, USA), and E-cadherin was from BD Biosciences (San Jose, CA, USA). Anti-Cdx1 and Cdx2 polyclonal antibodies were from cell signaling (Cell Signaling Technology, Inc. Danvers, MA). Antibody against GATA4 and the horseradish peroxidase-conjugated anti-rabbit and anti-goat antibodies were from Santa Cruz Biotechnology, Inc. (Santa Cruz, CA). The human Cdx1, Cdx2, GATA4 expression vectors (pRC/CMV-Cdx1, pRC/CMV-Cdx2, pCDNA3.1-GATA4 and pBJ5hHNF-1α) and Cdx2 truncation mutants (Flag-CD, Flag-δ15, Flag−/δ55-136, Flag-δ50, Flag-δ140-180) were generous gift from Dr. John P. Lynch (University of Pennsylvania, Philadelphia, PA). The activated β-catenin (constitutively active, CA) S33Y mutant and dominant negative (dn)-TCF-4 expression plasmids were provided by Dr. K.W. Kinzler (Johns Hopkins, Baltimore, MD) and Dr. H. Clevers (Netherlands Institute for Developmental Biology, Upsalalaan, Utrecht, the Netherlands) respectively. The claudin-1 luciferase reporter plasmid is described previously [21].

### Cell Culture and Transfection

The human colon cancer cell lines HCT116 and SW480 cells were obtained from ATCC and cultured in RPMI 1640 containing 10% FBS, 100 U/ml penicillin, and 100 µg/ml streptomycin. The renal tubular epithelial cells, MDCK-II cells and human breast cancer cells, MCF-7 were (from ATCC) grown in Dulbecco’s modified Eagle’s medium:nutrient mix F12 (Invitrogen, Carlsbad, CA) containing 10% FBS, 100 U/ml penicillin, and 100 µg/ml streptomycin. One day before transfection, cells were seeded in 6-well cell culture plates to provide a final density of 60–70% confluence. Cells were transfected using Effectene Transfection Reagent (QIAGEN Inc. USA) as described previously [21]. Forty hours after transfection, cells were harvested and luciferase activity was measured using the dual-luciferase reporter assay system (Promega, Madison, WI) and an Optocomp II Luminometer (MGM Instruments, Inc., Hamden, CT). Transfection efficiency was normalized to *Renilla* luciferase activity of the phRL-TK (Promega) and the results are expressed as the mean relative luciferase activity ± S.D. (at least three independent experiments). We calculated fold stimulation for each sample by dividing the normalized luciferase activity by the value obtained from the control transfection containing empty vector (pGL3-basic).

### RNA Isolation, Semiquantitative Reverse Transcription–PCR and Real-time PCR

Total RNA isolation and reverse transcription–PCR was performed using standard protocols as described earlier [5], [21]. For real-time PCR, complementary DNA (cDNA) was synthesized and PCR was carried out using SYBR PCR master kit (Applied Biosystems Inc., Foster City, CA, USA). The sequences of the claudin-1 primers are 5^′^-TCACTCCCAGGAGGATGC-3^′^ (Forward Primer) and 5^′^-GGCAGATCCAGTGCAAAGTC-3′ (Reverse Primer). PCR was carried out in triplicates for each gene being validated.

### Western Blot Analysis

Immunoblotting was done using antigen-specific antibody using standard protocol, as described previously [5]. The signal was visualized with horseradish peroxidase-conjugated secondary antibodies using enhanced chemiluminescence (Amersham Biosciences, Piscataway, NJ).

### Chromatin Immunoprecipitation (ChIP) Assay

ChIP assay was done using EZ-ChIP assay kit, (Upstate, Millipore, Billercia, MA, USA) as per manufacturer’s protocol. In brief, precleared and sonicated chromatin from 5×10^7^ (formaldehyde-treated) SW480 cells was immunoprecipitated using anti-Cdx2 antibody. Normal rabbit IgG served as antibody control. The supernatant from the above reaction lacking the primary antibody was saved as total input of chromatin and was processed in the same way as the eluted immunoprecipitates, starting with the cross-linking reversal step. After processing, immunoprecipitated chromatin was amplified using human claudin-1-specific sense (5^′^-AAACCGGAGGGTGAGAGATAG-3^′^ ) and antisense primers (5^′^-TCGCAGGTGAGGACATGTAA-3^′^ ). GAPDH-specific primers served as negative controls. PCR products were analyzed by electrophoresis on 1.5% agarose gels in 0.5× TBE buffer.

### Statistical Analysis

Data is presented as means ± SD. Statistical significance was tested, when applicable, with the t-test (P≤0.05 for significance) and Bonferroni correction was applied for multiple testing.

## Supporting Information

Figure S1 **Cdx1, Cdx2 and GATA4–dependent regulation of claudin-1 luciferase reporter.** SW480 cells were transiently transfected with a 3.2-kb claudin-1-luciferase reporter plasmid along with Cdx1, Cdx2 and GATA4 expression vectors. Empty vector pGL3-basic was used for control purposes. Nonspecific plasmid DNA was used to maintain equal amounts of DNA in all transfection samples. Results are expressed in fold-activation of relative luciferase activity after normalization with *Renilla* activity from 3 independent experiments, and the values are expressed as means ± SD.
(TIFF)

Figure S2 **HNF-1α-dependent regulation of claudin-1 luciferase reporter.** SW480 cells were transiently transfected with a 1.2-kb claudin-1-luciferase reporter plasmid along with HNF-1α expression vector. Empty pGL3-basic vector was used for control purposes. Results are expressed in fold-activation of relative luciferase activity after normalization with *Renilla* activity from 3 independent experiments, and the values are expressed as means ± SD.
(TIFF)
