# Supplementary figures and images for: Caudal Homeobox Protein Cdx-2 Cooperates with Wnt Pathway to Regulate Claudin-1 Expression in Colon Cancer Cells
Source: PLoS One. 2012 Jun 15;7(6):e37174. doi: 10.1371/journal.pone.0037174 (PMC3376107; doi:10.1371/journal.pone.0037174)

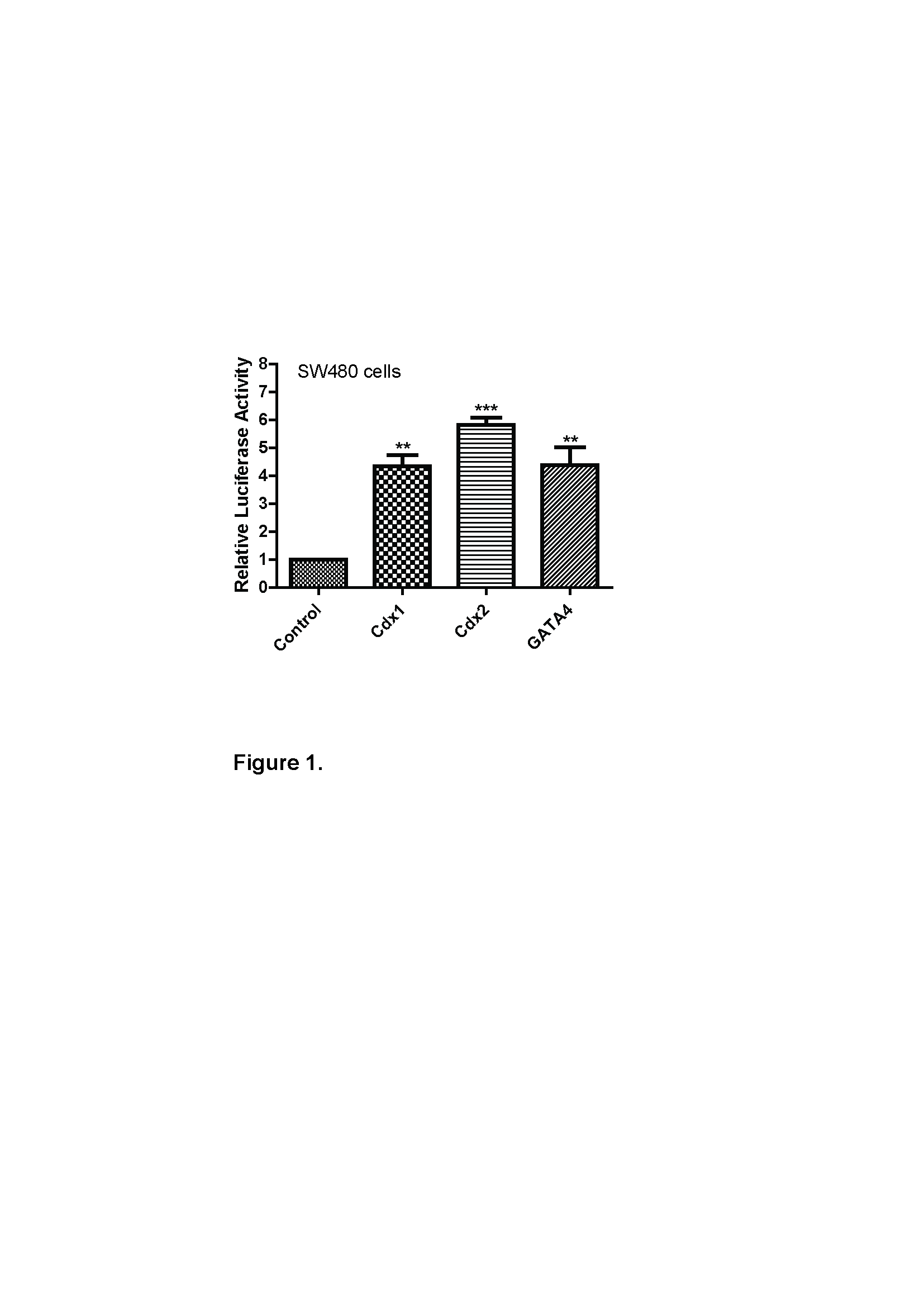

Supplement: Figure S1 — Cdx1, Cdx2 and GATA4–dependent regulation of claudin-1 luciferase reporter. SW480 cells were transiently transfected with a 3.2-kb claudin-1-luciferase reporter plasmid along with Cdx1, Cdx2 and GATA4 expression vectors. Empty vector pGL3-basic was used for control purposes. Nonspecific plasmid DNA was used to maintain equal amounts of DNA in all transfection samples. Results are expressed in fold-activation of relative luciferase activity after normalization with Renilla activity from 3 independent experiments, and the values are expressed as means ± SD. (TIFF) [file pone.0037174.s001.tif]

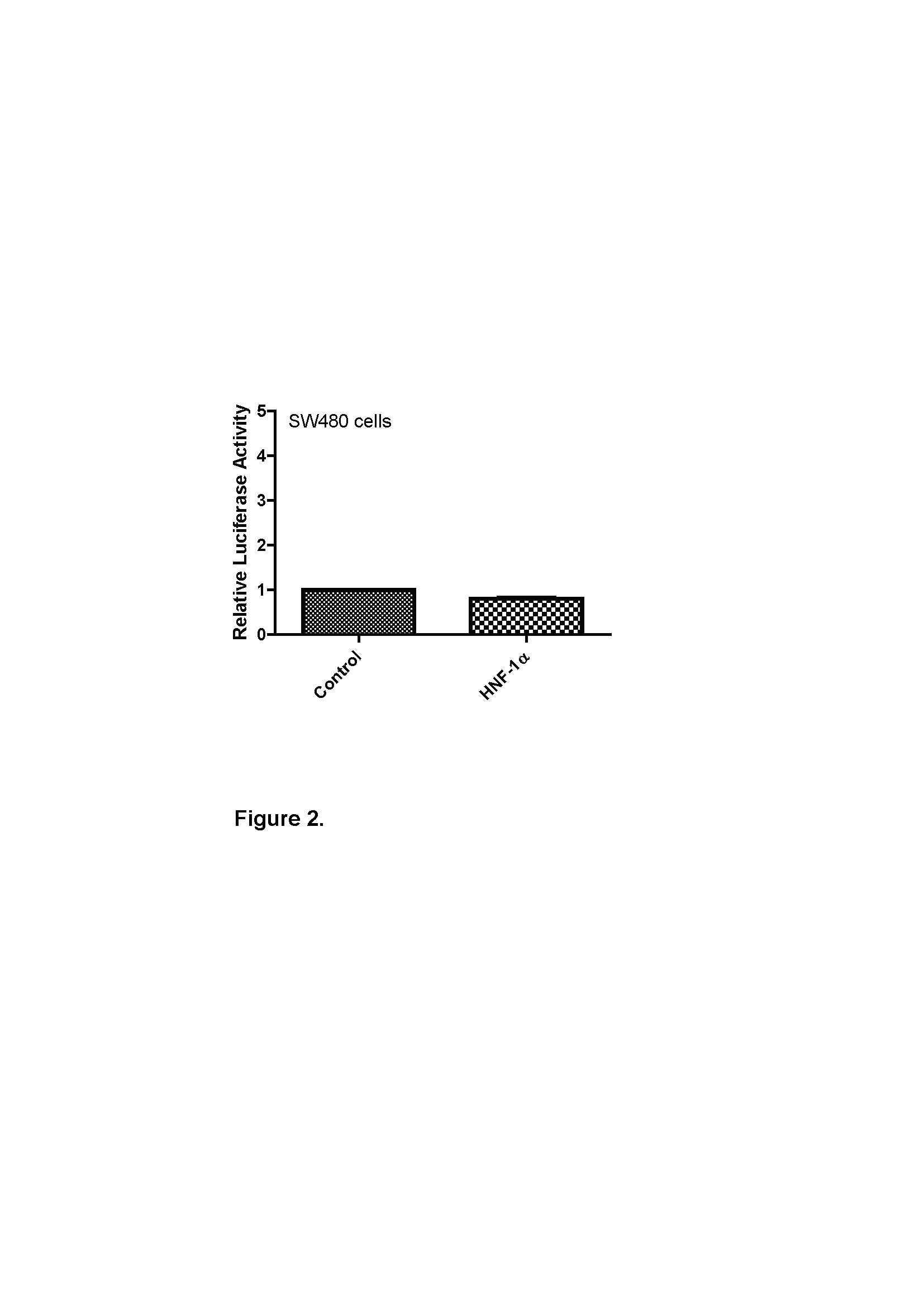

Supplement: Figure S2 — HNF-1α-dependent regulation of claudin-1 luciferase reporter. SW480 cells were transiently transfected with a 1.2-kb claudin-1-luciferase reporter plasmid along with HNF-1α expression vector. Empty pGL3-basic vector was used for control purposes. Results are expressed in fold-activation of relative luciferase activity after normalization with Renilla activity from 3 independent experiments, and the values are expressed as means ± SD. (TIFF) [file pone.0037174.s002.tif]
